# The dilemma of coastal management: Exploitation or conservation?

**DOI:** 10.1017/cft.2024.10

**Published:** 2024-08-14

**Authors:** M. Luisa Martínez, Rodolfo Silva, Octavio Pérez-Maqueo, Valeria Chávez, Gabriela Mendoza-González, Carmelo Maximiliano-Cordova

**Affiliations:** 1 Institute of Engineering, National Autonomous University of Mexico (UNAM), Mexico City, México; 2 Institute of Ecology, A.C. (INECOL), Xalapa, México; 3 National Laboratory of Sustainability Sciences (LANCIS), Institute of Ecology, ENES-Mérida, National Autonomous University of Mexico (UNAM), Mérida, México

**Keywords:** coastal dunes, tourism, storm protection, coastal management, ecosystem services, climate change, coastal squeeze, coastal armouring

## Abstract

The real estate business on sandy coasts and coastal dunes has increased dramatically over the last decades because of the growing demands for leisure activities which, consequently, have yielded important economic gains. Such ravaging exploitation results in the replacement of sandy ecosystems with tourism-oriented settlements, infrastructure, and facilities. As the sandy beaches and coastal dunes become deteriorated or eliminated, their protective role is lost, and the hydrometeorological risks to which the increasing human coastal populations are exposed grow, especially in a climate change scenario with increasing storminess. Furthermore, when possible, the expansion of the tourism industry continues searching for new, unspoiled locations, and the cycle begins again. This situation leads to the dilemma of coastal management: should we continue with the over-exploitation of sandy coasts for growing economic benefits? Or should we preserve the coasts for protection against the impact of increasing storms and sea level rise and to benefit biodiversity? Although scientific evidence demonstrates the relevance of protecting the coasts, coastal development plans continue to ignore these findings. What are the key drivers for these trends? We first looked for scientific evidence of the appraisal of the esthetic beauty of the beach and coastal dunes, as highly important drivers of urbanization and coastal environmental change. We then looked for evidence that demonstrated how coastal dunes offer storm protection Finally, we examined if the conservation of beaches and coastal dunes can be compatible with non-intrusive tourism. In summary, through the literature review and our own data, we show how different alternatives may help achieve a more sustainable coastal tourism by combining economic necessities with environmental concerns.

## Graphical abstract

**Figure:**
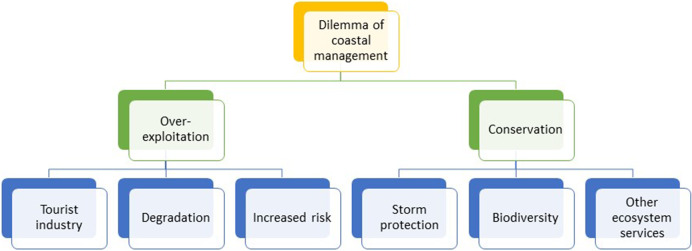

## Impact statement

This study explores the coastal management dilemma: should we continue with the over-exploitation of sandy beaches for coastal tourism and growing economic benefits? Or should we preserve the coasts for protection against the impact of increasing storms and sea level rise? To address this issue, we first looked for scientific evidence of the appraisal of the esthetic beauty of the beach and coastal dunes, as highly important drivers of urbanization and coastal environmental change. We then looked for evidence that demonstrated how coastal dunes offer storm protection. Finally, we examined different alternatives from the literature that can help achieve a more sustainable coastal tourism which helps combine economic necessities and environmental concerns.

## Introduction

Planet Earth is, without doubt, a coastal planet, with nearly 1.5 million km of oceanic coasts (The World Fact Book, 2020), which are highly heterogeneous and contain a wide variety of geomorphological features and ecosystems. Historically, the richness of natural resources and transportation possibilities on and along the coasts have provided significant trading and economic opportunities to society. Consequently, these regions have been attractive for human settlements for millennia (McGranahan et al., 2007; Silva et al., 2014), as seen in the number of cities along the coasts both now and in the past (Barragán and De Andrés, 2015). The number of people living in coastal cities is still increasing. McGranahan et al. (2007) estimated that the LECZ (Low Elevation Coastal Zones, located <10 m above sea level) represent approximately 2% of the total land surface of the world. However, 10% of the world’s human population live there. Indeed, many of the planet’s megacities (over 10 million inhabitants) are found near or on the coast (Martínez et al., 2007; Barragán and De Andrés, 2015).

Relevant socioeconomic activities take place in this rather narrow strip of land, of which tourism has become increasingly popular over the last decades, rendering significant economic revenues and intense development (Davenport and Davenport, 2006; Honey and Krantz, 2007; García Romero et al., 2022). The predominant sea, sand and sun tourism taking place on sandy beaches and coastal dunes is usually associated with the construction of large tourist complexes and facilities. The impact of tourism is manyfold. For instance, beaches in tourist destinations are raked for cleanliness (garbage and biological debris as well) (Mo et al., 2021), coastal dunes are flattened, and the plants are removed (Sytnik and Stecchi, 2015). These intensely urbanized coasts are abandoned by wild fauna (see, e.g., Jonah et al., 2015; Fantinato, 2019; Phillips et al., 2019). In all, the continuous influx of people generally results in loss or degradation of coastal ecosystems, resources and ecosystem services (see, e.g., Calderisi et al., 2021; Hogan et al., 2021; Mo et al., 2021). One of these services is the protection of human infrastructure and settlements (Keijsers et al., 2015; Gesing, 2019; Cunha et al., 2021; Mehrtens et al., 2023), which is increasingly necessary because of climate variability, climate change, and associated alterations in the marine environment (i.e., increased storminess, storm surges and sea level rise). Consequently, there are important economic losses and casualties in coastal settlements owing to extreme hydrometeorological events (Costanza et al., 2021; Martínez et al., 2023), and these are likely to increase in a climate change scenario. In fact, the Climate Change Index, calculated by Kreft et al. (2016), revealed that extreme weather events most affect countries on the coast, many of which are exposed to extreme weather events, such as tropical cyclones. Given these predictions, the protection of coastal settlements is becoming a critical issue worldwide.

Furthermore, as the tourist industry expands and environmental degradation increases, the once beautiful coasts become less attractive for humans and result in relevant economic losses due to decreased tourism and recovery costs. For instance, reef destruction (White et al., 2000), beach erosion (Flayou et al., 2021), beach litter (Ballance et al., 2000) or *Sargassum* blooms (Fraga and Robledo, 2022; Rodríguez-Martínez et al., 2023) all result in important economic losses because of reduced tourism and management costs to reverse degradation. That is, if environmental degradation is not prevented or attended, tourist visitation will decline which will likely lead to unemployment and bring social problems such as poverty and crime (Rodríguez-Martínez et al., 2023). Coastal tourism follows the “landscape-tourism cycle” in which tourist activity is driven by the expectations and interests of tourists (Tress and Tress, 2002). According to this landscape-tourism cycle, the landscapes attract tourists and influence their activities which, in turn, modify the landscape. However, tourist activity could not take place if the landscape cannot provide the basis for this (i.e., is altered or degraded). Furthermore, frequently, cities for coastal tourism oftentimes start as locations for the vacation of the elite and subsequently become the place for the amusement of working classes, leading to mass tourism (Nolasco‐Cirugeda et al., 2020; Lukoseviciute and Panagopoulos, 2021; Vitz, 2023). Then, the “landscape-tourism cycle” expands and starts somewhere else, where the original coastal landscape amusement is available.

In brief, although the need and relevance of protecting coastal ecosystems is becoming increasingly acknowledged by society, there is still an ongoing dilemma regarding land use decisions and the management of sandy beaches and coastal dunes. In a climate change scenario, with sea-level rise and increased storminess projections, it is worthy to reflect upon the following: should we continue expanding tourism thoughtlessly because it renders substantial economic benefits? Should we continue with abusive coastal over-exploitation for the tourist industry at the expense of environmental degradation? Or should we promote an alternative approach to tourism with the conservation of the beach and coastal dunes for protection from storms and sea-level rise? The answer may seem obvious: Protecting and preserving the coasts is highly relevant. Nevertheless, coastal development trends show that overexploitation is the most common decision. This is evident in the rampaging exploitation of the coasts (e.g., Mendoza-González et al., 2012; Seer et al., 2016; Phillips et al., 2019; Calderisi et al., 2021; Hogan et al., 2021; García Romero et al., 2022). So, it certainly is worthwhile to address this problem and highlight potential alternatives.

In this context, this study aims to address the coastal management dilemma: tourism exploitation versus conservation of beaches and coastal dunes. Is it possible to find a sustainable combination between these seemingly opposite alternatives? To face this challenge, we first looked for scientific evidence of tourism and the appraisal of the esthetic beauty of the beach and coastal dunes, as highly important drivers of urbanization and environmental change along the coastline. We then explored the other side of the coin: we looked for evidence that demonstrated how coastal dunes offer storm protection (especially when sand sources are healthy). Finally, we examined if the conservation of beaches and coastal dunes can be compatible with non-intrusive tourism which could help reach a dynamic balance between socioeconomic needs and environmental conservation.

## Methods

### Tourism and esthetic values

In August (2023), we searched for significant articles in the Web of Science database, with no restrictions on the year of publication. We used a simple combination of search strings: first, “coastal dune*” and then “tourism” OR “esthetic” OR “hedonic.” The goal was to find articles that focused on tourism or tourism-related activities taking place on sandy beaches and coastal dunes. Abstracts of the articles retrieved were read and, if relevant to this study, the full paper was analyzed. We looked for specific information in each article we read: the year of publication, the country where the study took place, the approach used to study tourism, and the methods followed. We also re-analyzed the results of two case studies, performed in Mexico, in which tourism and scenic beauty were addressed.

#### Case studies in Mexico

**Figure 1.**
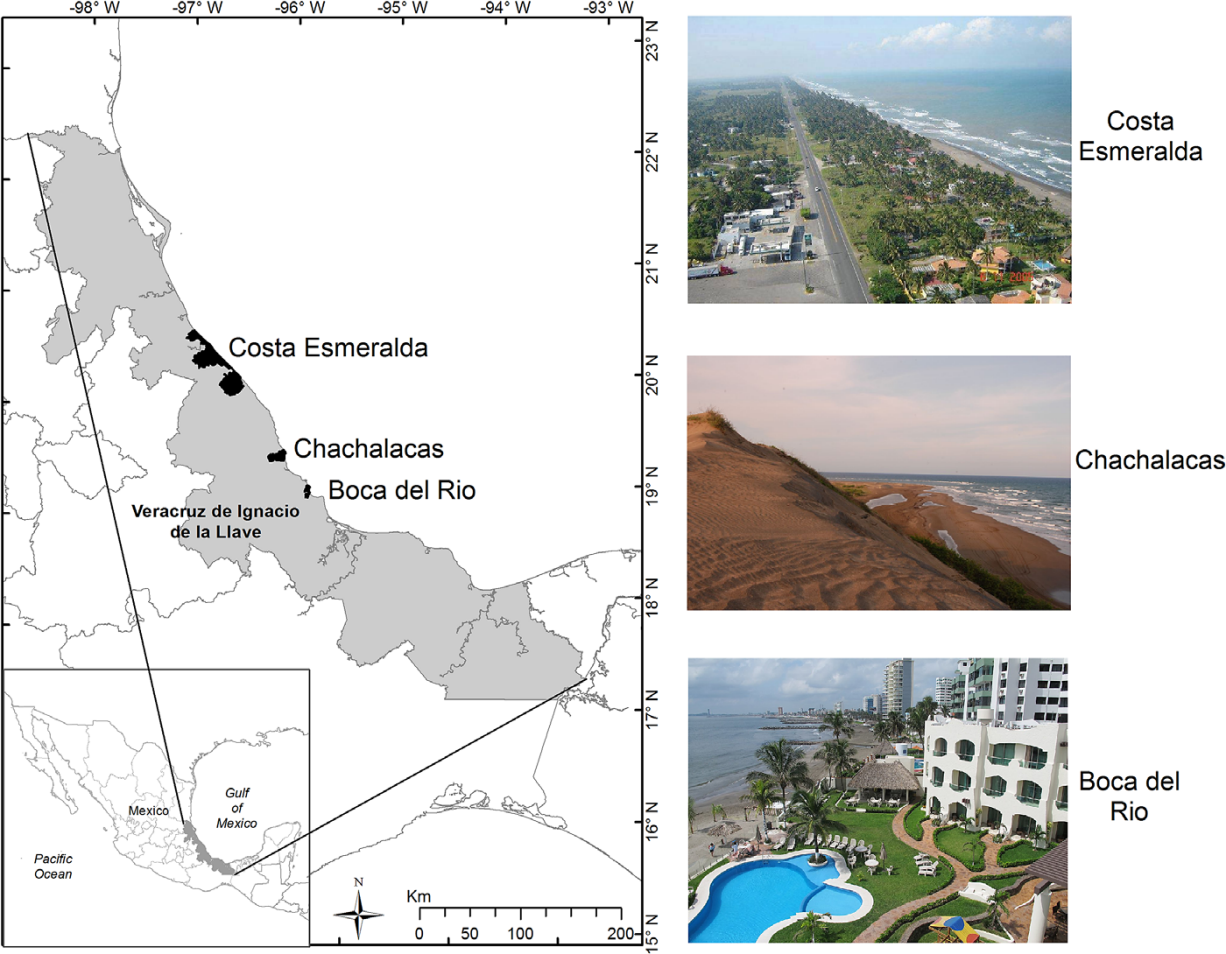
Location of the study sites where the studies on tourism and coastal dunes took place in Mexico. (Picture credits: Costa Esmeralda, Secretaria de Turismo y Cultura, Veracruz; Chachalacas, Mario Paredes, Flickr; Boca del Río, Emiliano Vázquez, Wikimedia).

For a more detailed example of tourism and coastal dunes, we re-analyzed and summarized two case studies from Mexico, which were carried out by some of the authors of this article. Both took place along the coast of Veracruz, on the Gulf of Mexico (Figure 1).

In the first case study, Mendoza-González et al. (2018) explored the value of ocean view and proximity to the beach in three locations, each with varying degrees of urbanization. They focused on two ecosystem services associated with tourism: esthetic (ocean view) and recreational (proximity to the beach). These authors looked for room prices at every hotel in a 300 m strip perpendicular to the coast, as at this distance tourists have most opportunity to access the recreational activities of the beach. Information was collected through interviews with hotel managers to whom we asked for room prices, with and without an ocean view. The georeferenced hotels were mapped and thus it was determined if the hotels had direct access to the beach or not. When hotel guests did not have to walk across a road, the hotel was considered as “close” to the beach; otherwise, they were grouped as being “far from the beach.” The number of hotels at each location varied, from the most urbanized, Boca del Río, with 16 hotels, to the moderately urbanized of Costa Esmeralda, with 50 hotels and, finally, Chachalacas (26 hotels), the least urbanized (Figure 1).

In the second study, Pérez-Maqueo et al. (2017) explored whether tourism and conservation can be compatible, rather than opposite activities. The study was carried out along the Costa Esmeralda region located along the central portion of the state of Veracruz (Figure 1). Here, beaches with different levels of infrastructure for tourism were found: site 1 had natural ecosystems, without any infrastructure; site 2 had a trailer park and site 3 had several low-density hotels and coastal facilities for tourists.

At each site, and at the time of day with highest tourist density (12–4 pm), photographs covering the whole width of the beach were shot every 100 m until the entire area where there were tourists was photographed. The photographs were taken at all the sites on the same day, no more than 30 min apart, and under similar weather conditions (bright sunny days), so that the number of tourists was not affected by the weather. Then, the beach area occupied by visitors was measured on site with a 50 m long measuring tape, showing that the photographs covered 3,000, 4,000 and 3,000 m^2^ at sites 1, 2 and 3, respectively. Later, the number of people seen in the photographs from each site was counted and the mean tourist density was calculated.

The spatial and temporal changes in vegetation cover, composition, and diversity were analyzed in 2008, at the three beaches before and after each high tourism season: Winter (December), Spring (April) and Summer (August). At each site, four randomly placed transects were set perpendicular to the shoreline. In each, 35 adjacent 2 × 2 m plots were placed from the top of the dune to the edge of the seaward vegetation. The transects and plots were marked to allow repetitive observations in the same location. A list of plant species was made for each plot and then the percentage cover per species was estimated visually. Vegetation sampling was always performed by the same person to minimize bias in the estimations. We used a database of the Flora of Veracruz for the identification of the species.

### Storm protection

In August (2023), we searched for significant articles in the Web of Science database, with no restrictions on the year of publication. We used a simple combination of search strings: first, “coastal dune” and then “storm protection.” The goal was to find articles that documented evidence of the storm protection value provided by coastal dunes. Abstracts of the articles retrieved were read and, if relevant to this study, the full paper was examined in greater detail. We looked for specific information in each article read: the year of publication, the country where the study took place, the disturbance regime that the dunes were protecting the coast against, and the type of evidence given of the protection provided by the dunes.

#### Case studies in Mexico

We summarized the results of different wave flume experiments that we have performed in Mexico (by some of the authors of the current study) to test the effectiveness of dunes and vegetation for protection against different storm intensities. In this paper, we compare the results of these experiments, something never done before, the only exception being Maximiliano-Cordova et al. (2023), where the morphological performance of coastal dunes under storm conditions was compared for vegetated and unvegetated dunes with a rocky versus a geotextile core. All the experiments compared here were carried out in a wave flume (0.8 m wide, 1.2 m high and 37 m long) at the Engineering Institute of the National Autonomous University of Mexico (IIUNAM). This flume has a piston-type wavemaker and is equipped with a dynamic wave absorption system. In the above-mentioned experiments, the channel was divided longitudinally, with a 1 cm thick acrylic sheet, thus allowing two conditions to be tested simultaneously. The dune profiles were built with sand from a beach where coastal dunes form naturally in northern Veracruz.

The experimental conditions had different beach and dune profiles, varying plant cover, varying species and species combinations, and the presence of a rocky core, as summarized in Table 1. The plant species used grow naturally on the beaches and coastal dunes of the Gulf of Mexico. Branches of these plants were cut on site, and then grown in green-house conditions until they were transplanted to the dunes built inside the wave-flume.

**Table 1.** Summary of experimental setups and conditions of the wave-flume experiments and the field observation study used here to test the protective role against dune erosion provided by the presence of plants

Experimental set-up	References
Two beach profiles, the presence *of Ipomoea pes–caprae* (low, medium and high density), three storm conditions	Silva et al., 2016
One beach profile, three plant species (*Ipomoea pes–caprae*, *Sesuvium portulacastrum*, *Sporobolus virginicus*), various combinations of species and one storm condition	Maximiliano–Cordova et al., 2019
One beach profile, presence of *Ipomoea pes–caprae* on two parts of the dune, rocky core and three storm conditions	Odériz et al., 2020
Three beaches, beach profile, vegetation cover before and after a winter storm	Maximiliano–Cordova et al., 2021

In addition to the laboratory experiments described above, there is another field study in which storm-induced erosion of coastal dunes was compared between dunes with varying plant cover, from vegetated to non-vegetated conditions. These field observations took place at three beaches in the Costa Esmeralda area. The beach profiles and vegetation cover, before and after a winter storm, were assessed in three transects running perpendicular to the shoreline of each beach (Maximiliano-Cordova et al., 2021). A list of plant species and the percentage cover of each was assessed in 2 × 2 m plots set along the transects. More detailed methods are found in Table 1 and in the original studies.

## Results

### Tourism and esthetic values

We found 3,185 studies on Web of Science that mentioned “coastal dune” in the title, abstract or keywords. Of these, 119 also mentioned tourism, 12 esthetic, 1 hedonic, and 7 scenic. After reading all abstracts, 75 studies were considered to directly address tourism on sandy beaches and coastal dunes, and were, therefore, analyzed in greater detail (see Supplementary Table 1 for detailed information on these findings).

**Figure 2.**
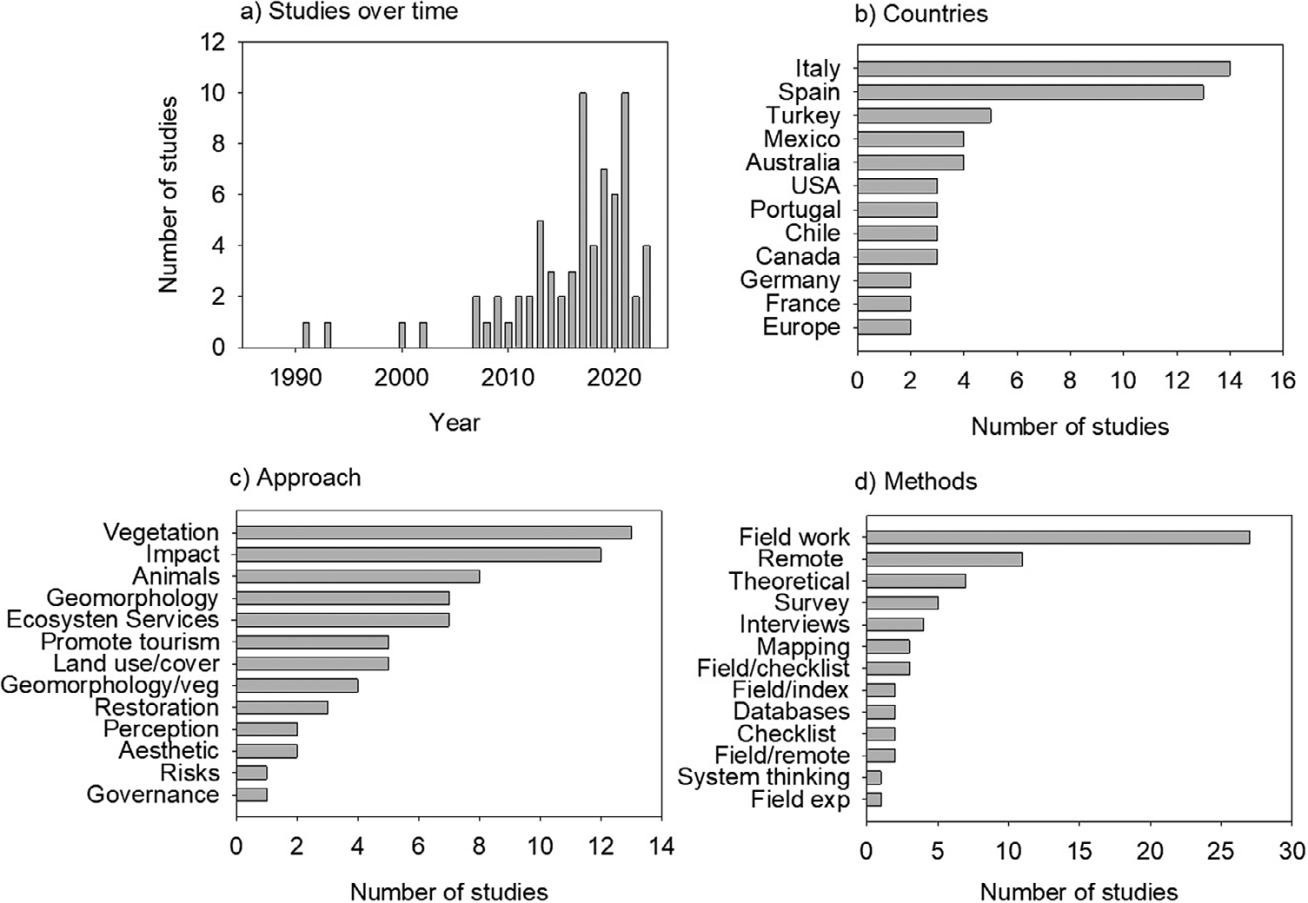
Summary of the findings of the literature review performed through Web of Science during August 2023 to explore the studies on coastal dunes and tourism. (a) Number of studies over time; (b) Countries where the studies took place (Note that those with only one study were not plotted for clarity -see text); (c) Approach used to study tourism and (d) Methods followed.

Our findings from the literature review show that there are few studies focused on tourism on coastal dunes, but that this number has increased recently, especially in the last decade (Figure 2a). Studies on this topic were carried out in 24 countries (Figure 2b), although in many of them, there was only one study (Algeria, Brazil, Ghana, Lithuania-Russia, Montenegro, Montenegro and Albania, Morrocco, South Africa, South Korea, South-Africa). These were not plotted for graphical clarity given their very low values. Spain, Italy and Turkey, which are among the most popular tourist destinations in the world, lead the list of publications, followed by Mexico, Chile and Australia. Different perspectives in studying tourism on coastal dunes were seen, although many focus on how tourism affects plants, environmental impacts, animal species or geomorphology (Figure 2c). Others explored how to promote tourism, or analyzed ecosystem services, or the esthetic value of the beach and coastal dunes. The focus least often mentioned concerns changes in land use/land cover, altered geomorphology and plant cover (combined), restoration actions, perception, risk and governance. Finally, we found that several methods are used in the studies on coastal dunes tourism of which field observations and remote sensing are the most frequently used (Figure 2d).

#### Case studies in Mexico

Mendoza-González et al. (2018) found that the prices of hotel rooms were significantly higher in hotels closer to the beach than in those located at a distance, across a road. In brief, the mean price of hotel rooms increased by 50 to 55% in hotels closer to the beach (Table 1). Similarly, these authors report that hotels and hotel rooms with an ocean view had higher prices than those without such view, in all the sites studied. Hotel rooms with an ocean view increased most in the most urbanized site (Boca del Río) and the increase was least in the more rural sites (Table 2).

**Table 2.** Percentage increase in hotel room prices in three study sites (2016 prices), associated with proximity to the beach (near vs. far) and access to an ocean view (view vs. no view)

Location	Type of location	% Increase Near versus Far	% Increase View versus No view
Boca del Río	Urban	54	43
Costa Esmeralda	Semi–urban	55	37
Chachalacas	Rural	50	20

*Note:* Data re-analyzed from Mendoza-González et al. (2018).

**Figure 3.**
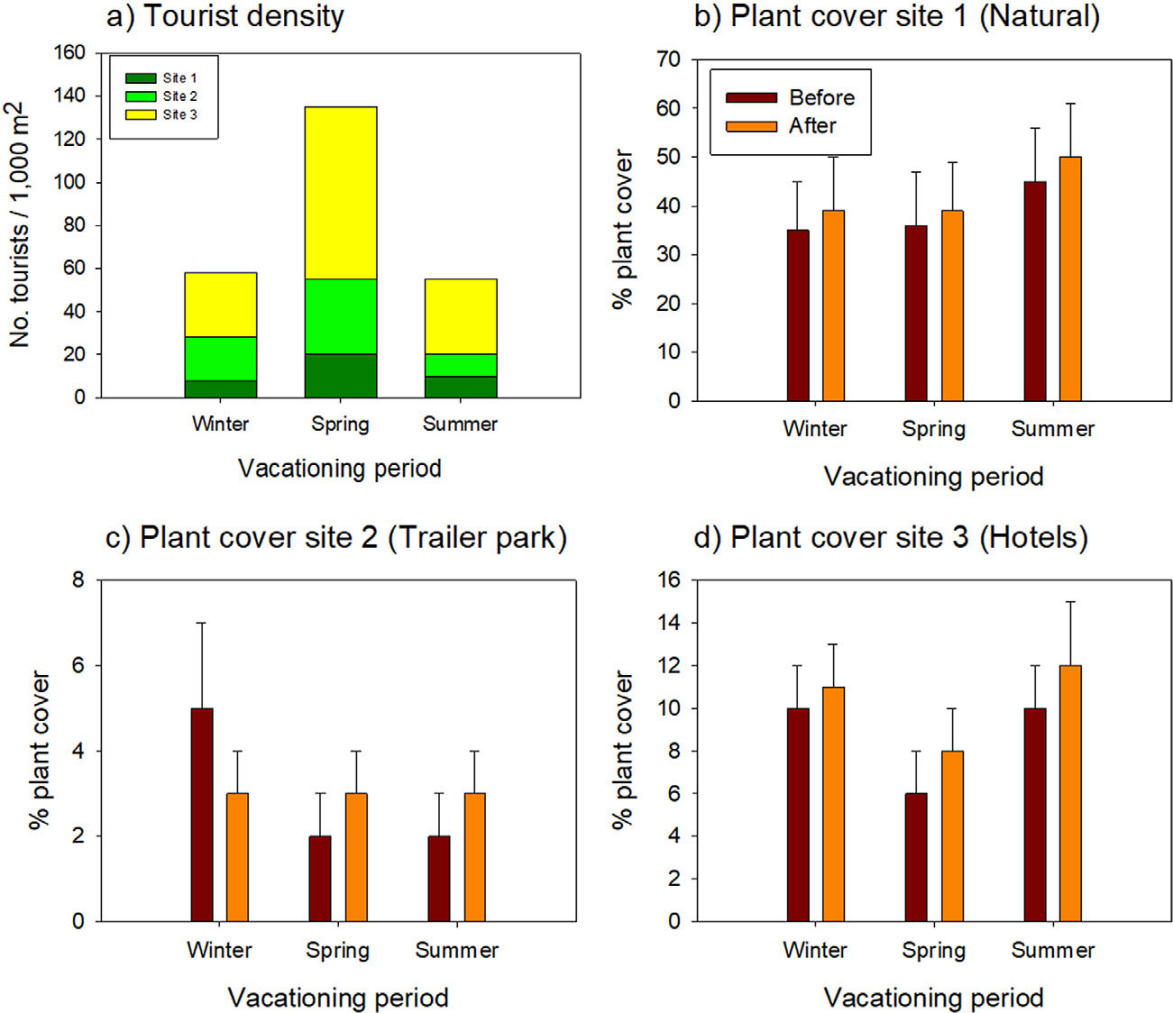
Tourist density and mean plant cover in three beach tourist destinations with different predominant land uses, before and after three high tourism seasons. (a) Tourist density in the three sites and during three tourist seasons; (b–d) Plant cover in each site with different conditions before and after the tourism season. Lines above the bars indicate standard deviations. Data modified from Pérez-Maqueo et al. (2017).

Pérez-Maqueo et al. (2017) reported that tourist density increased with the amount of infrastructure, from the most natural to the most abundant hotels. Because the study sites are in the tropic, the weather is mild throughout the year, and vacationing density depends more on the vacationing periods than on the weather. In this case, Pérez-Maqueo et al. (2017) found that the number of tourists was greatest in the Spring (Figure 3a). Contrary to what was expected, plant cover did not vary significantly before and after the peak tourist seasons (Figure 3b–d) at the three study sites. In fact, it increased slightly (but not significantly) in all sites, except for Winter at site 2, when plant cover decreased after the vacations. It is interesting that plant cover was highest at site 1, the site with the most natural conditions.

### Storm protection

Of the 3,185 studies found on Web of Science and mentioning “coastal dune*” in the title, abstract or keywords, only 68 mentioned “storm protection.” After reading the abstracts, 23 directly addressed the protective role of coastal dunes. Thus, these studies were analyzed in greater detail.

**Figure 4.**
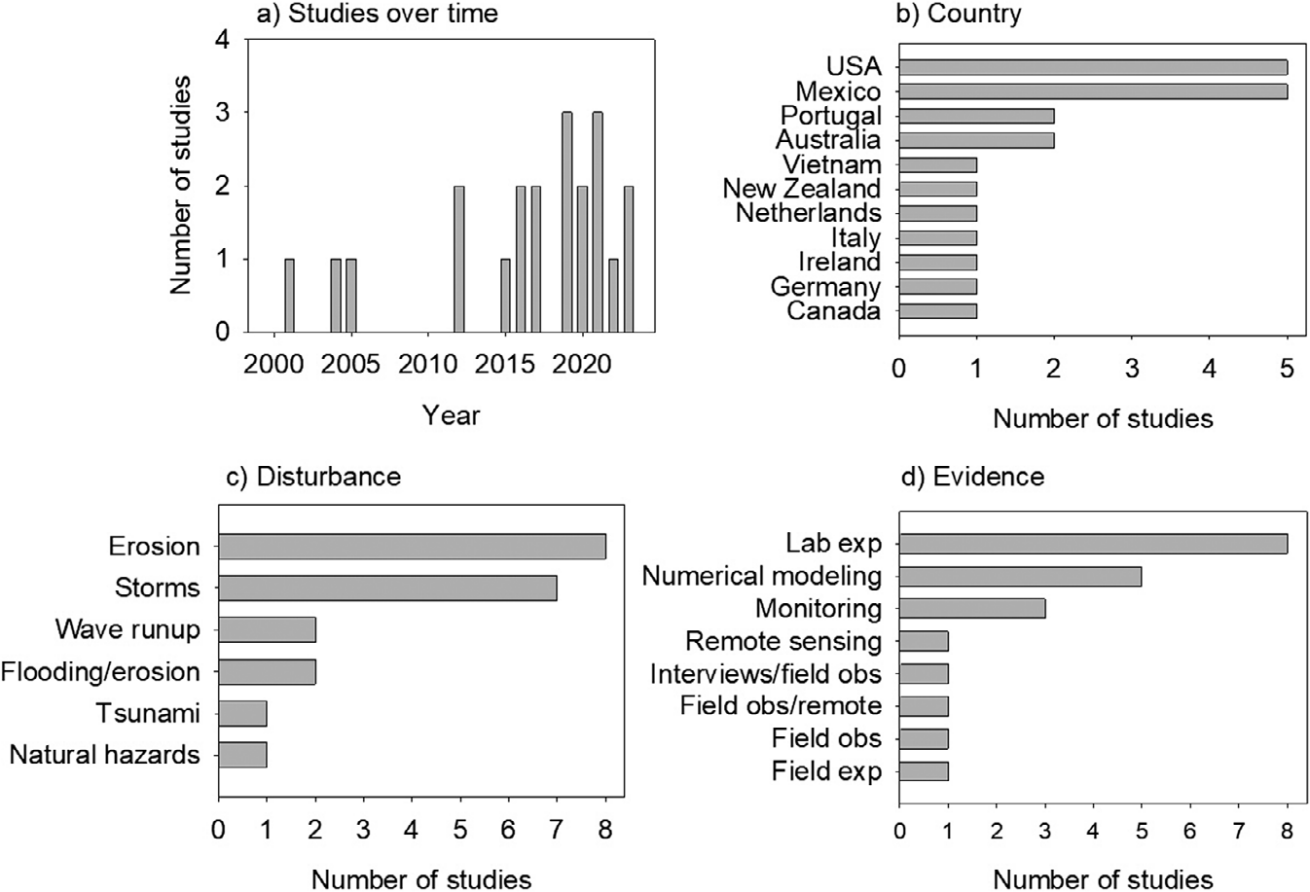
Summary of the findings of the literature review performed through Web of Science during August 2023 to explore the studies on the protective role of coastal dunes. (a) Number of studies over time; (b) Countries where the studies took place; (c) Disturbance from which coastal dunes protected and (d) Type of evidence to confirm protection.

Like the findings on tourism, the number of studies that focus on the storm protection provided by coastal dunes has increased recently, but the numbers are still very low (Figure 4a). Storm protection studies were performed in 11 countries (Figure 4b), although in many cases, there was only one study. The USA and Mexico had the largest number of studies, followed by Portugal and Australia. Various disturbances were considered, but storm impact and erosion were explored most (Figure 4c). Finally, the most frequent evidence are laboratory experiments, numerical modeling, and monitoring (Figure 4d). Very few studies used remote sensing, field observations or field experiments (Figure 4d; see Supplementary Table 2 for detailed information on these findings).

#### Case studies in Mexico

There is growing evidence of a decrease in erosion rates on vegetated dunes compared to dunes without vegetation, both in experimental and natural conditions (Table 3). In laboratory conditions, and under different experimental set-ups, erosion was reduced by 28 to 68% when there was plant cover on dunes. This trend was also seen in natural conditions, but the amount of reduction was much smaller (15%), and this pattern was only seen at one of the three sites. In this case, local conditions such as beach type, dune height, and the dominant plant species were important in the rate of erosion, besides plant cover.

**Table 3.** Summary of comparisons of studies on testing the protective role of vegetated dunes against storm-induced erosion

Experimental conditions	Erosion with plants	Erosion without plants	% difference (reduced erosion)	Reference
With a berm	75 cm^2^	130 cm^2^	58	Silva et al., 2016
Without a berm	70 cm^2^	150 cm^2^	47	Silva et al., 2016
*Ipomoea* one species	0.02 m^3^	0.04 m^3^	50	Maximiliano–Cordova et al., 2019
*Sesuvium* one species	0.02 m^3^	0.04 m^3^	50	Maximiliano–Cordova et al., 2019
*Ipo–Sporo–Ses* combined	0.022 m^3^	0.04 m^3^	55	Maximiliano–Cordova et al., 2019
Storm 1 with *Ipomoea*	1.47%	5.12%	28	Odériz et al., 2020
Storm 2 with *Ipomoea*	2.90%	8.08	36	Odériz et al., 2020
Storm 3 with *Ipomoea*	3.90	5.72	68	Odériz et al., 2020
Natural plant cover (field obs)	0.1 m^3^ (high plant cover)	0.65 m^3^ (low plant cover)	15	Maximiliano–Cordova et al., 2021

In brief, the results from these studies indicate that (a) vegetation reduced erosion; (b) vegetation modified the type of erosion, and overwash only occurred when there was no vegetation; (c) the berm was important in reducing erosion; and (d) dune resilience increased with plant cover. However, the authors report that their results were not linear and depended on the combination of beach and dune profile, storm intensity and plant architecture.

## Discussion

The dilemma of exploitation versus conservation of coastal management has been acknowledged for a long time (see, for instance, the literature reviews by Jones, 2022; Vitz, 2023). On one hand, the intense tourist activities that lead to urbanization and economic growth are important drivers for land use change and environmental degradation. On the other, the need to protect and restore rapidly decaying beaches and coastal dunes is urgent if we are to recover the resilience of these ecosystems as well as important ecosystem services such as storm protection, scenic beauty, and recreation (Everard et al., 2010). It is, therefore, necessary to explore whether the benefits of coastal dunes and sandy beaches for tourism can be enjoyed without damaging the ecological structure and functioning of these natural ecosystems. In the following sections, we discuss the expansion of coastal tourism and the academic studies in this regard. We then analyze the evidence of the protective role provided by coastal dunes and, in both cases identify the gaps in the current knowledge. Finally, we explore the potential actions that can help face the coastal management dilemma.

### The tsunami of coastal tourism

Without a doubt, tourism is a very important industry, worldwide. In 2019, before the COVID-19 pandemic, global tourism provided jobs for 82.5 million people and the direct contribution of travel and tourism to the global GDP was 19% (USD 1,653 billion) (https://www.unwto.org/tourism-statistics/tourism-statistics-database). Coastal tourism is one of the largest segments of the tourist industry, and the fastest-growing in terms of job opportunities and economic importance (Papageorgiou, 2016). Coastal tourism seems like a socioeconomic tsunami. In 2021 it was estimated that the coastal and maritime tourism market size was USD 2.9 trillion, and it is expected to grow by 5.7% from 2022 to 2030 (https://www.grandviewresearch.com/industry-analysis/coastal-maritime-tourism-market-report). Many coastal countries are mass tourism destinations, particularly in areas with tropical and Mediterranean climates. In their worldwide analysis, Onofri and Nunes (2013) report that the coastal countries with most international coastal arrivals are Spain (with 33 million per year), followed by Italy (32), the USA (30) and France (22). According to these authors, the variables that correlated the most with these international coastal arrivals were beach lengths and UNESCO cultural heritage sites (Onofri and Nunes, 2013), which highlights the relevance of the beach for coastal tourism.

These global trends in coastal tourism have increasingly captured the attention of academics. We saw in the literature review that in the last decade an increasing number of scientific articles have explored tourism on sandy beaches and coastal dunes. However, the number of countries where these studies were performed is very limited; only 24 of the 160 countries with a coastline (Onofri and Nunes, 2013), representing 15% of the total found. Most of the studies deal with the impacts of tourism on the vegetation, geomorphology, and animals. But relatively few explored the possibility of combining tourism and conservation.

### The protective role of coastal dunes

As the human population increases along the coasts (McGranahan et al., 2007) and natural hazards (storms and sea level rise) become more extreme, the protection of coastal settlements has become a socioeconomic priority. However, there is no consensus on the best way to protect our coasts (Cooper and McKenna, 2008). To some, this means building structures designed to halt flooding and coastal erosion and protect property, while to others it means allowing coastal ecosystems to function naturally and conserve their dynamics, while infrastructure and population are translated landwards (Phillips and Jones, 2006). The outcomes of these alternatives are contrasting. A hard-protected coastline modifies sediment and hydrodynamic flows and so the erosion problems often move downdrift (Chávez et al., 2021). In turn, when coasts are shaped by natural processes, they can respond to environmental fluctuations. Nowadays the creation and restoration of natural ecosystems on the coast is considered as a cost-effective means of protection which is both sustainable and ecologically sound (Temmerman et al., 2013), with the benefit of multiple ecosystem services such as scenic beauty, recreation, water quality and habitat. However, a nature-based protection needs time and space to work, and this is not always suitable to modern fast-moving societies.

There is still limited evidence of the protective role of coastal dunes against storm impact and erosion, but it is increasing. Coastal dunes with varying levels of plant cover have been shown to be more efficient at dissipating wave energy and thus, reducing erosion. However, most of this evidence comes from laboratory experiments. Consequently, given the many shapes of coastal dunes and varying plant species that grow on them, further evidence is still necessary, using a variety of methods: laboratory experiments, numerical modeling, and field observations. More abundant and conclusive evidence would make it possible to advocate the use of nature-based coastal protection measures more strongly, which also offer socioeconomic and ecological benefits.

### Combining tourism with conservation: Ten examples of alternatives

The current pressures and future challenges faced by the coasts are neither simple nor linear (Barbier et al., 2008), due to their varying ecological and socioeconomic characteristics and needs, coupled with the inherent and constantly evolving complexity of the coastal environment (Moser et al., 2012). According to the landscape-tourism cycle (Tress and Tress, 2002), the landscape provides different functions and meanings to contemporary tourists: a place for recreation and emotion; for culture; habitat to visit or to live in. These activities and functions are interwoven with the environment, the physical dimension of the landscape. Thus, as the landscape changes and deteriorates, so do these associated functions. Specifically, the disorderly tourism development is setting this industry, the coastal environment, and coastal settlements at risk. Indeed, the accumulating evidence of the environmental impacts of coastal tourism clearly indicates that we should continue exploring how to maintain this very significant economic activity, without destroying the very essence of it: the scenic beauty and recreation possibilities. This means that the current coastal tourism industry cannot be sustained indefinitely and thus, we need to think at a meta-level and include other parameters (such as environmental degradation) before reaching a no-return condition (Rangel-Buitrago et al., 2018).

Indeed, as many tourists prioritize being very near the beach to better enjoy the scenic beauty and recreation, they are willing to pay a significant premium for this (Mendoza-González et al., 2018), for instance, hotel prices. These priorities, in turn, trigger further tourist infrastructure development on the coast, and thus, the ocean view becomes limited by the towering hotel complexes, making the beach and ocean view less accessible. And, perhaps more worryingly, ecosystem-based coastal protection is lost. As the sandy beaches and coastal dunes become deteriorated (and thus, less attractive for tourists), the “discerning tourist,” and the “astute investor” search for new, unspoiled locations, and the cycle begins again (Tress and Tress, 2002; Arabadzhyan et al., 2021; Vitz, 2023). Therefore, decision-makers and coastal managers increasingly must reconcile the multiple uses of the coast with the protection of beaches and coastal dunes. In this sense, robust data on the type, extent, and magnitude of impacts of tourism, for example, are critical to formulate efficient management strategies for sandy beaches and coastal dunes.

Certainly, the problem is complex, and many factors need to be considered to solve it. Some examples of alternatives and conditions are mentioned in Table 4 elaborated from the literature review. The list of recommendations is not exhaustive but provides 10 good examples of necessary actions. These span a broad array of alternatives that include maintaining the dynamics of natural processes (for instance sediment transport), considering climate change and coastal squeeze, promoting ecotourism, and respecting ecosystem thresholds, while assessing trade-offs between ecosystem services. Furthermore, it would be beneficial if varying development actions were considered, such as deploying infrastructure adequately, making nature-based long-term plans for urbanization and land use changes and, of course, considering the perspective of tourists and local inhabitants (Table 4).

**Table 4.** Examples from the literature review show actions that help combine tourism with nature protection

Recommendation	Description	Example	References
Maintain natural physical processes	The maintenance of natural dynamics, and adaptation to them, should be part of the coastal management plan of areas with tourist development	Latin America	Silva et al., 2014
Assess the impact of climate change	Physical, ecological, and socio–economic impacts of climate change should be anticipated. These differ across destinations	Global approach	Arabadzhyan et al., 2021
Consider the impact of coastal squeeze	If tourist destinations undergo coastal squeeze and are in flood–prone sites, the infrastructure is in high–risk conditions	Mexico	Lithgow et al., 2019
Promote ecotourism	New forms of tourism, such as the many options of ecotourism, are more sustainable, will help to improve the human relationship with the environment and the host societies	Caribbean islands	Theng et al., 2014
Combine ecosystem thresholds with tourism	Medium–density tourism can be compatible with the protection of beach and coastal dune vegetation	Mexico/Australia	Pérez–Maqueo et al., 2017; Phillips et al., 2017
Consider ecosystem services perspective	The trade–offs between various ecosystem services for nature–based tourism should be evaluated	Mediterranean coast	Drius et al., 2019
Deploy adequate infrastructure	The use of boardwalks could be an effective tool for reducing the negative impact of bathing tourism on coastal dunes and requires little, inexpensive maintenance	Italy	Fantinato, 2019; Prisco et al., 2021
Make long–term urban plans to maintain environmental quality	Inadequate urban planning leads to overcrowding and environmental degradation	Spain	García-Ayllón, 2015; Nolasco‐Cirugeda et al., 2020
Develop sustainable land use plans	Urban expansion in coastal regions can lead to dramatic land–use changes, which can natural ecosystems and accelerate the decrease in coastal resilience	South Korea	Kim et al., 2017
Bear in mind the perspective of the tourist	The attractions seen by the tourists will help to focus on providing opportunities for conserving natural resources while offering opportunities for recreation	Portugal	Lukoseviciute and Panagopoulos, 2021

More conclusive scientific evidence is needed to explore how tourism can be compatible with a reduced environmental impact. Can we reach a dynamic equilibrium between socioeconomic needs and ecological priorities, that would lead to more resilient and healthier coasts? The literature reveals different paths that could help moving coastal development and management toward this end. Finally, on top of finding this balance between tourism activities and species conservation, it is important to bear in mind that natural and preserved areas (untouched by humans as much as possible) are still necessary. Coastal management actions should also prioritize the protection of the coastal biodiversity and the many species that cannot thrive in human-modified environments.

## Conclusions: Future perspectives

In this study we aimed to explore solutions to the coastal management dilemma: on one hand, the expanding sea, sand, and sun tourism industry produce very relevant economic benefits, especially because of the beautiful ocean view and recreation possibilities at the coast. But this leads to extensive ecosystem loss and degradation. On the other hand, the protective role of coastal dunes is lost as these are degraded or destroyed, and in consequence, the risk to which the increasing human coastal populations are exposed grows especially in a climate change scenario. Furthermore, degraded coasts are less attractive for tourists and thus, tourists and investors search for new, unspoiled locations, and the cycle begins again. In this context, we need to think about the coastal management dilemma. Should we push the economic benefits of sea, sand, and sun tourism at the expense of environmental degradation and increasing risks for coastal settlements? Or is it time to rethink coastal management and change the paradigm of the tourism industry toward the transition of human coexistence with nature? This would slow down the environmental degradation of coastal tourism without sacrificing it.

The future perspectives of coastal management need to further explore if we can reach a dynamic equilibrium between socioeconomic needs and desires with ecological priorities, that would lead to more resilient and healthier coasts. The coastal management dilemma can be solved not by choosing between either tourism or conservation, but by exploring different options that include, among others, the physical processes, consider the impact of climate change and coastal squeeze, promote ecotourism alternatives, combine ecosystem thresholds with the economy, consider ecosystem services, and promoting nature-based urbanization plans. Finally, we should not disregard the relevance of preserving species and ecosystems away from the impact of tourism, since many of them are not compatible with human activities.

One final note: as we finished writing this study, the city of Acapulco, on the Mexican Pacific, was hit on October 24, 2023 by Otis, a category 5 hurricane. Because of abnormally warm temperature on the ocean surface, Otis developed from tropical storm to hurricane category 5 within a few hours and directly hit this very relevant coastal touristic city with 800,000 inhabitants plus thousands of tourists (50% occupancy at the time Otis made landfall). Certainly, there is no natural ecosystem that could have avoided the dreadful damages caused by 350 km/h gusts of winds. But perhaps, as Acapulco is rebuilt (hopefully very soon), we can think of better ways of doing so by improving construction norms, window frames (most of them damaged), and restoring degraded ecosystems, so that this iconic coastal city is even more beautiful than before Otis. Acapulco is a very sad example of why now, more than ever, coastal cities need to be prepared for the unexpected.

## Supporting information

Martínez et al. supplementary material 1 Martínez et al. supplementary material

Martínez et al. supplementary material 2 Martínez et al. supplementary material

## Data Availability

Data used in this are supplied in the Supplementary Tables and further details will be available upon request to the corresponding author.

## Supplementary material

The supplementary material for this article can be found at http://doi.org/10.1017/cft.2024.10.
