# Supplementary material for: The dilemma of coastal management: Exploitation or conservation?
Source: Camb Prism Coast Futur. 2024 Aug 14;2:e10. doi: 10.1017/cft.2024.10 (PMC12337577; doi:10.1017/cft.2024.10)
Supplement: Martínez et al. supplementary material 1 — Martínez et al. supplementary material [file S2754720524000106sup001.docx]

Suplemmentary material 1. Literature review of scientific studies focused on tourism and coastal dunes.

| Year | Authors | Approach | Methods | Country |
| --- | --- | --- | --- | --- |
| 1991 | Guilcher and Hallégouët, 1991 | Impact | Theoretical | France |
| 1993 | Watkeys et al., 1993 | Promote tourism | Theoretical | South-Africa |
| 2000 | Nordstrom et al., 2000 | Promote tourism | Theoretical | USA |
| 2002 | Catto, 2002 | Geomorphology | Theoretical | Canada |
| 2007 | Grunewald and Schubert, 2007 | Impact | Field work/checklist | Germany |
| 2007 | Sönmez and Sarİ, 2007 | Land use/cover | Remote sensing | Turkey |
| 2008 | Comor et al., 2008 | Animals | Field work | France |
| 2009 | Kemal Sönmez et al., 2009 | Land use/cover | Remote sensing | Turkey |
| 2009 | Özcan et al., 2009 | Promote tourism | Mapping | Turkey |
| 2010 | Everard et al., 2010 | Ecosysten Services | Theoretical | Europe |
| 2011 | Schlacher et al., 2011 | Animals | Field work | Australia |
| 2011 | Vallés et al., 2011 | Geomorphology/veg | Checklist | Spain |
| 2012 | El Mrini et al., 2012 | Geomorphology | Field work | Morrocco |
| 2012 | Yang et al. 2012 | Promote tourism | Remote sensing | USA |
| 2013 | Bessa et al., 2013 | Animals | Field work | Portugal |
| 2013 | Cabrera-Vega et al. 2013 | Geomorphology | Remote sensing | Spain |
| 2013 | Ballantyne and Pickering, 2013 | Vegetation | Databases | Europe |
| 2013 | Farris et al., 2013 | Vegetation | Field work | Italy |
| 2013 | Attorre et al., 2013 | Vegetation | Field work | Italy |
| 2014 | Bessa et al., 2014 | Animals | Field work | Portugal |
| 2014 | Pintó et al., 2014 | Geomorphology/veg | Field work/checklist | Spain |
| 2014 | Lucrezi et al., 2014 | Vegetation | Field work | South Africa |
| 2015 | Jonah et al., 2015 | Animals | Field work | Ghana |
| 2015 | Sytnik and Stecchi, 2015 | Geomorphology/veg | Published info, field, remote | Italy |
| 2016 | Seer et al., 2016 | Impact | Field work | Germany |
| 2016 | Teixeira et al., 2016 | Restoration | Field experiment | Brazil |
| 2016 | Martinez et al., 2016 | Vegetation | Field work | Mexico |
| 2016 | Šilc et al., 2016 | Vegetation | Field work | Montenegro and Albania |
| 2017 | Phillips et al., 2017 | Animals | Field work | Australia |
| 2017 | Carpio et al., 2017 | Animals | Field work | Spain |
| 2017 | Ferrer-Valero et al., 2017 | Geomorphology | Mapping | Spain |
| 2017 | González and Holtmann-Ahumada, 2017 | Impact | Field/index | Chile |
| 2017 | Ciccarelli et al., 2017 | Impact | Field/index | Italy |
| 2017 | Hernández-Cordero et al., 2017 | Land use/cover | Remote sensing | Spain |
| 2017 | Kim et al., 2017 | Land use/cover | System thinking process | South Korea |
| 2017 | Pérez-Maqueo et al., 2017 | Vegetation | Field work | Mexico |
| 2017 | Gómez-Zotano et al., 2017 | Vegetation | Field work remote sensing | Spain |
| 2018 | Carranza et al., 2018 | Ecosysten Services | Mapping | Italy |
| 2018 | Bezzi et al., 2018 | Geomorphology | Databases | Italy |
| 2018 | Garcia-Lozano and Pintó, 2018 | Land use/cover | Remote sensing | Spain |
| 2019 | Urbis et al., 2019 | Aesthetic | Survey | Lithuania |
| 2019 | Fantinato, 2019 | Animals | Field work | Italy |
| 2019 | Drius et al., 2019 | Ecosysten Services | Interviews | Italy |
| 2019 | Senouci and Taibi, 2019 | Geomorphology | Remote sensing | Algeria |
| 2019 | Hogan et al., 2019 | Impact | Interviews | Canada |
| 2019 | Lithgow et al., 2019 | Risks | Remote sensing | Mexico |
| 2019 | Pinna et al., 2019 | Vegetation | Field work | Italy |
| 2019 | Phillips et al., 2019 | Impact | Field work | Australia |
| 2020 | Carranza et al., 2020 | Ecosysten Services | Survey | Italy |
| 2020 | Harris et al., 2020 | Ecosysten Services | Survey | USA |
| 2020 | Lithgow et al., 2020 | Restoration | Field work/checklist | Mexico |
| 2020 | San Nayim, 2020 | Vegetation | Field work | Turkey |
| 2020 | Šilc et al., 2020 | Vegetation | Field work | Montenegro |
| 2021 | Urbis et al., 2021 | Aesthetic | Remote sensing | Lithuania-Russia |
| 2021 | Arévalo-Valenzuela et al., 2021 | Ecosysten Services | Survey | Chile |
| 2021 | Santana-Cordero, 2021 | Geomorphology/veg | Remote sensing | Spain |
| 2021 | Bellaubi et al., 2021 | Governance | Theoretical | Spain |
| 2021 | Calderisi et al., 2021 | Impact | Field work | Italy |
| 2021 | Prisco et al., 2021 | Impact | Field work | Italy |
| 2021 | Mo et al., 2021 | Impact | Field work | Italy |
| 2021 | Hogan et al., 2021 | Impact | Interviews | Canada |
| 2021 | González et al., 2021 | Perception | Survey | Chile |
| 2022 | García-Romero et al., 2022 | Impact | Field work | Spain |
| 2022 | Pinna et al., 2022 | Restoration | Checklist | Italy |
| 2023 | Doyle and Woodroffe, 2023 | Geomorphology | Remote sensing | Australia |
| 2023 | Gül and Küçükuysal 2023 | Promote tourism | Field work | Turkey |
| 2023 | Enriquez de Salamanca, 2023 | Vegetation | Field work | Spain |
| 2023 | Pintó et al., 2023 | Vegetation | Field work | Spain |

**References**

**Arévalo-Valenzuela P, Peña-Cortés F and Pincheira-Ulbrich J** (2021) Ecosystem services and uses of dune systems of the coast of the Araucania Region, Chile: A perception study. *Ocean & Coastal Management*, **200**(105450), 1–11. doi:https://doi.org/10.1016/j.ocecoaman.2020.105450.

**Attorre F, Maggini A, Di Traglia M, De Sanctis M and Vitale, M** (2013) A methodological approach for assessing the effects of disturbance factors on the conservation status of Mediterranean coastal dune systems. *Applied Vegetation Science*, **16**(2), 333–342. doi:10.1111/avsc.12002.

**Ballantyne M and Pickering CM** (2013) Tourism and recreation: A common threat to IUCN red-listed vascular plants in Europe. *Biodiversity and Conservation*, **22**(13–14), 3027–3044. doi:10.1007/s10531-013-0569-2.

**Bellaubi F, Mallarach JM and Sardá R** (2021) A geoethical approach to unlock a social-ecological governance problem: The case of the tordera river (Catalonia, Spain). *Sustainability*, **13**(4253), 1–14. doi:10.3390/su13084253.

**Bessa F, Cunha D, Gonçalves SC and Marques, JC** (2013) Sandy beach macrofaunal assemblages as indicators of anthropogenic impacts on coastal dunes. *Ecological Indicators*, **30**, 196–204. doi:https://doi.org/10.1016/j.ecolind.2013.02.022.

**Bessa F, Goncalves SC, Franco JN, André JN, Cunha PP and Marques, JC** (2014) Temporal changes in macrofauna as response indicator to potential human pressures on sandy beaches. *Ecological Indicators*, **41**, 49–57. doi:https://doi.org/10.1016/j.ecolind.2014.01.023.

**Bezzi A, Pillon S, Martinucci D and Fontolan G** (2018) Inventory and conservation assessment for the management of coastal dunes, Veneto coasts, Italy. *Journal of Coastal Conservation*, **22**(3), 503–518. doi:10.1007/s11852-017-0580-y.

**Cabrera-Vega LL, Cruz-Avero N, Hernández-Calvento L, Hernández-Cordero AI and Fernández-Cabrera E** (2013) Morphological changes in dunes as an indicator of anthropogenic interferences in arid dune fields. *Journal of Coastal Research*, **65**(2), 1271–1276. doi:10.2112/si65-215.1.

**Calderisi G, Cogoni D, Pinna MS and Fenu G** (2021) Recognizing the relative effects of environmental versus human factors to understand the conservation of coastal dunes areas. *Regional Studies in Marine Science*, **48**(102070), 1–9. doi:https://doi.org/10.1016/j.rsma.2021.102070.

**Carpio AJ, Figueras M and Tortosa FS** (2017) Walkway on coastal dunes negatively affects mobility of the spiny-footed lizard acanthodactylus erythrurus. *Animal Biodiversity and Conservation*, **40**(2), 159–164. doi:10.32800/abc.2017.40.0159.

**Carranza ML, Drius M, Malavasi M, Frate L, Stanisci A and Acosta ATR** (2018) Assessing land take and its effects on dune carbon pools. An insight into the Mediterranean coastline. *Ecological Indicators*, **85**, 951–955. doi:10.1016/j.ecolind.2017.10.052.

**Carranza ML, Drius M, Marzialetti F, … Stanisci A** (2020) Urban expansion depletes cultural ecosystem services: an insight into a Mediterranean coastline. *Rendiconti Lincei.Scienze Fisiche e Naturali*, **31**(1), 103–111. doi:10.1007/s12210-019-00866-w.

**Catto N** (2002) Anthropogenic pressures on coastal dunes, southwestern Newfoundland. *Canadian Geographer/Le Géographe Canadien*, **46**(1), 17–32. doi:https://doi.org/10.1111/j.1541-0064.2002.tb00728.x.

**Ciccarelli D, Pinna MS, Alquini F, … Fenu G** (2017) Development of a coastal dune vulnerability index for Mediterranean ecosystems: A useful tool for coastal managers? *Estuarine, Coastal and Shelf Science*, **187**, 84–95. doi:https://doi.org/10.1016/j.ecss.2016.12.008.

**Comor V, Orgeas J, Ponel P, Rolando C and Delettre YR** (2008) Impact of anthropogenic disturbances on beetle communities of French Mediterranean coastal dunes. *Biodiversity and Conservation*, **17**(8), 1837–1852. doi:10.1007/s10531-007-9290-3.

**Doyle TB and Woodroffe CD** (2023) Modified foredune eco-morphology in southeast Australia. *Ocean & Coastal Management*, **240**, 106640.

**Drius M, BongiorniL, Depellegrin, D, Menegon S, Pugnetti A and Stifter S** (2019) Tackling challenges for Mediterranean sustainable coastal tourism: An ecosystem service perspective. *Science of the Total Environment*, **652**, 1302–1317. doi:10.1016/j.scitotenv.2018.10.121.

**El Mrini A, Anthony EJ, Maanan M, Taaouati M and Nachite D** (2012) Beach-dune degradation in a Mediterranean context of strong development pressures, and the missing integrated management perspective. *Ocean & Coastal Management*, **69**, 299–306. doi:https://doi.org/10.1016/j.ocecoaman.2012.08.004.

**Enriquez de Salamanca Á** (2023) Synanthropisation of coastal vegetation in southern Spain. *Mediterranean Botany*, (44), 1–14. doi:https://doi.org/10.5209/mbot.78628.

**Everard M, Jones L and Watts B** (2010) Have we neglected the societal importance of sand dunes? An ecosystem services perspective. *Aquatic Conservation: Marine and Freshwater Ecosystems*, **20**(4), 476–487. doi:10.1002/aqc.1114.

**Fantinato E** (2019) The impact of (mass) tourism on coastal dune pollination networks. *Biological Conservation*, **236**, 70–78. doi:https://doi.org/10.1016/j.biocon.2019.05.037.

**Farris E, PisanuS, Ceccherelli G and Filigheddu R** (2013) Human trampling effects on Mediterranean coastal dune plants. *Plant Biosystems-an International Journal Dealing with All Aspects of Plant Biology*, **147**(4), 1043–1051. doi:https://doi.org/10.1080/11263504.2013.861540.

**Ferrer-Valero N, Hernández-Calvento L and Hernández-Cordero AI** (2017) Human impacts quantification on the coastal landforms of Gran Canaria Island (Canary Islands). *Geomorphology*, **286**, 58–67. doi:10.1016/j.geomorph.2017.02.028.

**Garcia-Lozano C and Pintó J** (2018) Current status and future restoration of coastal dune systems on the Catalan shoreline (Spain, NW Mediterranean Sea). *Journal of Coastal Conservation*, **22**(3), 519–532. doi:10.1007/s11852-017-0518-4.

**García-Romero L, Peña-Alonso C, Hesp PA, Hernández-Cordero AI and Hernández-Calvento L** (2022) Sand, Sun, Sea and Sex with Strangers, the “five S’s”. Characterizing “cruising” activity and its environmental impacts on a protected coastal dunefield. *Journal of Environmental Management*, **301**(113931), 1–13. doi:10.1016/j.jenvman.2021.113931.

**Gómez-Zotano J, Olmedo-Cobo JA and Arias-García J** (2017) Mediterranean dune vegetation: conservation of a threatened ecosystem in southern Spain. *Geografisk Tidsskrift - Danish Journal of Geography* , **117**(1), 36–52. doi:10.1080/00167223.2016.1267579.

**González SA and Holtmann-Ahumada G** (2017) Quality of tourist beaches of northern Chile: A first approach for ecosystem-based management. *Ocean & Coastal Management*, **137**, 154–164. doi:https://doi.org/10.1016/j.ocecoaman.2016.12.022.

**González SA, Loyola D and Yañez-Navea K** (2021) Perception of environmental quality in a beach of high social segregation in northern Chile: Importance of social studies for beach conservation. *Ocean & Coastal Management*, **207**(105619), 1–14. doi:https://doi.org/10.1016/j.ocecoaman.2021.105619.

**Grunewald R and Schubert H** (2007) The definition of a new plant diversity index “H′ dune” for assessing human damage on coastal dunes—Derived from the Shannon index of entropy H′. *Ecological Indicators*, **7**(1), 1–21. doi:https://doi.org/10.1016/j.ecolind.2005.09.003.

**Guilcher A and Hallégouët B** (1991) *Coastal Dunes in Brittany and Their Management* *Journal of Coast Research*, Vol. 7.

**Gül M and Küçükuysal C** (2023) Geotourism activities via marine excursion: Muğla, SW Türkiye. *Geoheritage*, **15**(64), 1–22. doi:10.1007/s12371-023-00830-z.

**Harris E, Schmutz PP, Jackson CA, Anaya M, Johnson M and Chapman G** (2020) Beach User Perception of the Economic and Ecological Services of Sand Dunes at Pensacola Beach, Florida. *Southeastern Geographer*, **60**(4), 309–331. doi:10.1353/sgo.2020.0026.

**Hernández-Cordero AI, Hernández-Calvento L and Espino EP-C** (2017) Vegetation changes as an indicator of impact from tourist development in an arid transgressive coastal dune field. *Land Use Policy*, **64**, 479–491. doi:https://doi.org/10.1016/j.landusepol.2017.03.026.

**Hogan JL, Sponarski CC and Bath AJ** (2019) All-terrain vehicles: Differences in perceptions of impact on coastal dunes among communities in New Brunswick, Canada. *Journal of Outdoor Recreation and Tourism*, **26**, 61–71. doi:https://doi.org/10.1016/j.jort.2019.02.001.

**Hogan JL, Sponarski CC and Vaske JJ** (2021) All-terrain vehicle use: A value-norm-perception model predicting perceived impact on coastal dunes. *Journal of Outdoor Recreation and Tourism*, **35**(100300), 1–6. doi:https://doi.org/10.1016/j.jort.2020.100300.

**Jonah FE, Aheto DW, Adjei-Boateng D, Agbo NW, Boateng I and Shimba MJ** (2015) Human use and modification of beaches and dunes are linked to ghost crab (Ocypode spp) population decline in Ghana. *Regional Studies in Marine Science*, **2**, 87–94. doi:https://doi.org/10.1016/j.rsma.2015.08.013.

**Kemal Sönmez N, Onur I, Sari M and Maktav D** (2009) Monitoring changes in land cover/use by CORINE methodology using aerial photographs and IKONOS satellite images: A case study for Kemer, Antalya, Turkey. In *International Journal of Remote Sensing*, Vol. 30, Taylor and Francis Ltd., , 1771–1778. doi:10.1080/01431160802639723.

**Kim M, You S, Chon J and Lee J** (2017) Sustainable land-use planning to improve the coastal resilience of the social-ecological landscape. *Sustainability*, **9**(1086), 2–21. doi:10.3390/su9071086.

**Lithgow D, Martínez ML, Gallego-Fernández JB, Pérez-Maqueo O and Silva R** (2020) Assessing the current state and restoration needs of the beaches and coastal dunes of Marismas Nacionales, Nayarit, Mexico. *Ecological Indicators*, **119**(106859), 1–8. doi:10.1016/j.ecolind.2020.106859.

**Lithgow D, Martínez ML, Gallego-Fernández JB, Silva R and Ramírez-Vargas DL** (2019) Exploring the co-occurrence between coastal squeeze and coastal tourism in a changing climate and its consequences. *Tourism Management*, **74**, 43–54. doi:10.1016/j.tourman.2019.02.005.

**Lucrezi S, Saayman M and van der Merwe P** (2014) Influence of infrastructure development on the vegetation community structure of coastal dunes: Jeffreys Bay, South Africa. *Journal of Coastal Conservation*, **18**(3), 193–211. doi:10.1007/s11852-014-0307-2.

**Martinez ML, Silva R, Mendoza E, Odériz I and Pérez-Maqueo O** (2016) Coastal dunes and plants: An ecosystem-based alternative to reduce dune face erosion. In *Journal of Coastal Research*, Vol. 1, Coastal Education Research Foundation Inc., , 303–307. doi:10.2112/SI75-061.1.

**Mo A, D’Antraccoli M, Bedini G and Ciccarelli D** (2021) The role of plants in the face of marine litter invasion: A case study in an Italian protected area. *Marine Pollution Bulletin*, **169**(112544), 1–10. doi:https://doi.org/10.1016/j.marpolbul.2021.112544.

**Nordstrom KF, Lampe R and Vandemark LM** (2000) Reestablishing Naturally Functioning Dunes on Developed Coasts. *Environmental Management*, **25**(1), 37–51. doi:doi.org/10.1007/s002679910004.

**Özcan H, Akbulak C, Kelmt A, Tosunoǧlu M and Uysal I** (2009) Ecotourism potential and management of kavak delta (Northwest Turkey). *Journal of Coastal Research*, **25**(3), 781–787. doi:10.2112/08-1068.1.

**Pérez-Maqueo O, Martínez ML and Cóscatl Nahuacatl R** (2017) Is the protection of beach and dune vegetation compatible with tourism? *Tourism Management*, **58**, 175–183. doi:10.1016/j.tourman.2016.10.019.

**Phillips VF, Chambers B and Bencini R** (2017) Body condition, breeding time and joey survival rates of the quokka (*Setonix brachyurus*) are improved in habitats developed for tourism on Rottnest Island, Western Australia. *Hystrix*, **28**(1), 48–55. doi:10.4404/hystrix-28.1-12186.

**Phillips VF, Chambers BK and Bencini R** (2019) Habitats modified for tourism affect the movement patterns of an endemic marsupial, the Rottnest Island quokka (*Setonix brachyurus*). *Australian Mammalogy*, **42**(1), 48–57. doi:https://doi.org/10.1071/AM17063.

**Pinna MS, Bacchetta G, Cogoni D and Fenu G** (2019) Is vegetation an indicator for evaluating the impact of tourism on the conservation status of Mediterranean coastal dunes? *Science of the Total Environment*, **674**, 255–263. doi:https://doi.org/10.1016/j.scitotenv.2019.04.120.

**Pinna MS, Cogoni D, Bacchetta G and Fenu, G** (2022) Assessing the potential for restoring Mediterranean coastal dunes under pressure from tourism. *Journal of Coastal Conservation*, **26**(15), 1–14. doi:10.1007/s11852-022-00860-9.

**Pintó J, Garcia-Lozano C and Varga D** (2023) Using dune-restricted species to assess the degree of natural diversity of dune systems on Mediterranean tourist coasts. *Ecological Indicators*, **147**(11004), 1–14. doi:https://doi.org/10.1016/j.ecolind.2023.110004.

**Pintó J, Martí C and Fraguell RM** (2014) Assessing current conditions of coastal dune systems of mediterranean developed shores. *Journal of Coastal Research*, **30**(4), 832–842. doi:10.2112/JCOASTRES-D-13-00116.1.

**Prisco I, Acosta ATR and Stanisci A** (2021) A bridge between tourism and nature conservation: boardwalks effects on coastal dune vegetation. *Journal of Coastal Conservation*, **25**(14), 1–14. doi:10.1007/s11852-021-00809-4.

**San Nayim Y** (2020) Assessment of sand dune ecosystems with Pancratium maritimum, Bartın, Turkey. *Environmental Biology*, **41**(2), 483–490. doi:10.22438/jeb/41/2(SI)/JEB-29.

**Santana-Cordero AM** (2021) Human-derived environmental consequences for three sedimentary systems of the canary islands (Spain) – a study of changes and impacts: A synthesis. *Geographical Research Letters*, **47**(2), 435–455. doi:10.18172/cig.4932.

**Schlacher TA, De Jager R and Nielsen T** (2011) Vegetation and ghost crabs in coastal dunes as indicators of putative stressors from tourism. *Ecological Indicators*, **11**(2), 284–294. doi:https://doi.org/10.1016/j.ecolind.2010.05.006.

**Seer FK, Irmler U and Schrautzer J** (2016) Beaches under pressure–effects of human access on vegetation at Baltic Sea beaches. *Applied Vegetation Science*, **19**(2), 225–234. doi:https://doi.org/10.1111/avsc.12221.

**Senouci R and Taibi N-E** (2019) Impact of the urbanization on coastal dune: case of Kharrouba, west of Algeria. *Journal of Sedimentary Environments*, **4**(1), 90–98. doi:10.12957/jse.2019.39951.

**Šilc U, Dajić Stevanović Z, Ibraliu A, Luković M and Stešević D** (2016) Human impact on sandy beach vegetation along the southeastern Adriatic coast. *Biologia (Poland)*, **71**(8), 865–874. doi:10.1515/biolog-2016-0111.

**Šilc U, Stešević D, Luković M and Caković D** (2020) Changes of a sand dune system and vegetation between 1950 and 2015 on Velika plaža (Montenegro, E Mediterranean). *Regional Studies in Marine Science*, **35**(101139), 1–9. doi:10.1016/j.rsma.2020.101139.

**Sönmez NK and Sarİ M** (2007) Monitoring land use change in the West Mediterranean region of Turkey: a case study on Antalya-Turkey coast. *Fresenius Environmental Bulletin*, **16**(11a), 1325–1330.

**Sytnik O and Stecchi F** (2015) Disappearing coastal dunes: tourism development and future challenges, a case-study from Ravenna, Italy. *Journal of Coastal Conservation*, **19**(5), 715–727. doi:10.1007/s11852-014-0353-9.

**Teixeira LH, Weisser W and Ganade G** (2016) Facilitation and sand burial affect plant survival during restoration of a tropical coastal sand dune degraded by tourist cars. *Restoration Ecology*, **24**(3), 390–397. doi:https://doi.org/10.1111/rec.12327.

**Urbis A, Povilanskas R, JurkusE, Taminskas, J and Urbis D** (2021) GIS-based aesthetic appraisal of short-range viewsheds of coastal dune and forest landscapes. *Forests*, **12**(1534), 1–23. doi:10.3390/f12111534.

**Urbis A, Povilanskas R and Newton A** (2019) Valuation of aesthetic ecosystem services of protected coastal dunes and forests. *Ocean and Coastal Management*, **179**(104832), 1–13. doi:10.1016/j.ocecoaman.2019.104832.

**Vallés SM, Gallego Fernández JB and Dellafiore CM** (2011) Dune vulnerability in relation to tourism pressure in Central Gulf of Cádiz (SW Spain), a case study. *Journal of Coastal Research*, **27**(2), 243–251. doi:10.2112/JCOASTRES-D-09-00125.1.

**Watkeys MK, Mason ZR and Goodman PS** (1993) The role of geology in the development of Maputaland, South Africa. *Journal of Afrlcan Earth Sciences*, **16**(1/2), 205–221.

**Yang B, Madden M, Kim J and Jordan TR** (2012) Geospatial analysis of barrier island beach availability to tourists. *Tourism Management*, **33**(4), 840–854. doi:https://doi.org/10.1016/j.tourman.2011.08.013.
