# Supplementary material for: The dilemma of coastal management: Exploitation or conservation?
Source: Camb Prism Coast Futur. 2024 Aug 14;2:e10. doi: 10.1017/cft.2024.10 (PMC12337577; doi:10.1017/cft.2024.10)
Supplement: Martínez et al. supplementary material 2 — Martínez et al. supplementary material [file S2754720524000106sup002.docx]

Suplemmentary material 2. Literature review of scientific studies focused on protection provided by coastal dunes.

| Year | Authors | Evidence | Disturbance | Country |
| --- | --- | --- | --- | --- |
| 2001 | Power et al., 2001 | Monitoring | Erosion | Ireland |
| 2004 | Forbes et al., 2004 | Monitoring | Erosion | Canada |
| 2005 | Reis et al., 2005 | Field exp | Storms | Portugal |
| 2008 | Pries et al., 2008 | Field obs | Storms | USA |
| 2012 | Seabloom et al., 2013 | Field obs/remote/modeling | Storms | USA |
| 2012 | Splinter and Palmsten, 2012 | Num modelling | Storms | Australia |
| 2015 | Keijsers et al., 2015 | Monitoring | Flooding/erosion | Netherlands |
| 2016 | Martínez et al., 2016 | Lab exp | Erosion | Mexico |
| 2016 | Silva et al., 2016 | Lab exp | Erosion | Mexico |
| 2017 | Charbonneau et al., 2017 | Field obs | Erosion | USA |
| 2017 | Mendoza et al., 2017 | Lab exp | Erosion | Mexico |
| 2017 | Fallon et al., 2017 | Field obs | Storms | USA |
| 2019 | Maximiliano-Cordova et al., 2019 | Lab exp | Erosion | Mexico |
| 2019 | Gesing, 2019 | Interviews/field obs | Natural hazards | New Zealand |
| 2019 | Feagin et al., 2019 | Lab exp | Erosion | USA |
| 2020 | Odériz et al., 2020 | Lab exp | Erosion | Mexico |
| 2020 | Johnson et al., 2020 | Remote/modelling | Storms | USA |
| 2021 | Maximiliano-Cordova et al., 2021 | Field obs | Storms | Mexico |
| 2021 | Cunha et al., 2021 | Num modelling | Storms | Portugal |
| 2021 | Dang et al., 2021 | Num modelling | Storms | Vietnam |
| 2021 | Innocenti et al., 2021 | Lab exp | Wave runup | USA |
| 2022 | Patel et al., 2022 | Lab exp | Tsunami | Australia |
| 2023 | Maximiliano-Cordova et al., 2023 | Lab exp | Erosion | Mexico |
| 2023 | Unguendoli et al., 2023 | Num modelling | Flooding/erosion | Italy |
| 2023 | Mehrtens et al., 2023 | Num modelling | Storms | Germany |

**References**

**Charbonneau BR, Wootton LS, Wnek JP, Langley JA and Posner MA** (2017) A species effect on storm erosion: Invasive sedge stabilized dunes more than native grass during Hurricane Sandy. *Journal of Applied Ecology*, **54**(5), 1385–1394. doi:10.1111/1365-2664.12846.

**Cunha J, Cardona FS, Bio A and Ramos S** (2021) Importance of Protection Service Against Erosion and Storm Events Provided by Coastal Ecosystems Under Climate Change Scenarios. *Frontiers in Marine Science*, **8**(726145), 1–15. doi:10.3389/fmars.2021.726145.

**Dang KB, Nguyen TT, Ngo HH, … Pham TPN** (2021) Integrated methods and scenarios for assessment of sand dunes ecosystem services. *Journal of Environmental Management*, **289**(January), 1–15. doi:10.1016/j.jenvman.2021.112485.

**Fallon AR, Hoagland P, Jin D, Phalen W, Fitzsimons GG and Hein CJ** (2017) Adapting without Retreating: Responses to Shoreline Change on an Inlet-Associated Coastal Beach. *Coastal Management*, **45**(5), 360–383. doi:10.1080/08920753.2017.1345607.

**Feagin RA, Furman M, Salgado K, … Silva R** (2019) The role of beach and sand dune vegetation in mediating wave run up erosion. *Estuarine, Coastal and Shelf Science*, **219**, 97–106. doi:https://doi.org/10.1016/j.ecss.2019.01.018.

**Forbes DL, Parkes GS, Manson GK and Ketch LA** (2004) Storms and shoreline retreat in the southern Gulf of St. Lawrence. *Marine Geology*, **210**(1–4), 169–204. doi:10.1016/j.margeo.2004.05.009.

**Gesing F** (2019) The politics of artificial dunes: Sustainable coastal protection measures and contested socio-natural objects. *DIE ERDE–Journal of the Geographical Society of Berlin*, **150**(3), 145–157. doi:https://doi.org/10.12854/erde-2019-423.

**Innocenti RA, Feagin RA, Charbonneau BR, … Smith J** (2021) The effects of plant structure and flow properties on the physical response of coastal dune plants to wind and wave run-up. *Estuarine, Coastal and Shelf Science*, **261**(August), 1–15. doi:10.1016/j.ecss.2021.107556.

**Johnson, CL Chen, Q and Ozdemir CE** (2020) Lidar time-series analysis of a rapidly transgressing low-lying mainland barrier (Caminada Headlands, Louisiana, USA). *Geomorphology*, **352**(106979), 1–23. doi:https://doi.org/10.1016/j.geomorph.2019.106979.

**Keijsers JGS, Giardino A, Poortinga A, Mulder JPM, Riksen MJPM and Santinelli, G** (2015) Adaptation strategies to maintain dunes as flexible coastal flood defense in The Netherlands. *Mitigation and Adaptation Strategies for Global Change*, **20**(6), 913–928. doi:10.1007/s11027-014-9579-y.

**Martinez ML, Silva R, Mendoz, E, Odériz I and Pérez-Maqueo O** (2016) Coastal dunes and plants: An ecosystem-based alternative to reduce dune face erosion. In *Journal of Coastal Research*, Vol. 1, Coastal Education Research Foundation Inc., , 303–307. doi:10.2112/SI75-061.1.

**Maximiliano-Cordova C, Martínez ML, Silva R, Hesp PA, Guevara R and Landgrave R** (2021) Assessing the Impact of a Winter Storm on the Beach and Dune Systems and Erosion Mitigation by Plants. *Frontiers in Marine Science*, **8**(734036), 1–20. doi:10.3389/fmars.2021.734036.

**Maximiliano-Cordova C, Salgado K, Martínez ML, … Feagin RA** (2019) Does the Functional Richness of Plants Reduce Wave Erosion on Embryo Coastal Dunes? *Estuaries and Coasts*, **42**(7), 1730–1741. doi:10.1007/s12237-019-00537-x.

**Maximiliano-Cordov, C, Silva R, Mendoza E, Chávez V, Martínez ML and Feagin RA** (2023) Morphological Performance of Vegetated and Non-Vegetated Coastal Dunes with Rocky and Geotextile Tube Cores under Storm Conditions. *Journal of Marine Science and Engineering*, **11**, 1–15. doi:10.3390/jmse11112061.

**Mehrten, B, Lojek O, Kosmalla V, Bölker T and Goseberg N** (2023) Foredune growth and storm surge protection potential at the Eiderstedt Peninsula, Germany. *Frontiers in Marine Science*, **9**(January), 1–21. doi:10.3389/fmars.2022.1020351.

**Mendoza E, Odériz I, Martínez ML and Silva R** (2017) Measurements and Modelling of Small Scale Processes of Vegetation Preventing Dune Erosion. *Journal of Coastal Research*, **Special Is**(77), 19–27. doi:10.2112/SI77-003.1.

**Odériz I, Knöchelmann N, Silva R, Feagin RA, Martínez ML and Mendoza, E** (2020) Reinforcement of vegetated and unvegetated dunes by a rocky core: A viable alternative for dissipating waves and providing protection? *Coastal Engineering*, **158**(February), 1–11. doi:10.1016/j.coastaleng.2020.103675.

**Patel DM, Wuppukondur A and Baldock TE** (2022) Experimental investigation of tsunami runup reduction in the presence of a coastal dune. In *Oceans Conference Record (IEEE)*, , 1–7. doi:10.1109/OCEANSChennai45887.2022.9775376.

**Power J, Cooper A, McKenna J, McGourty J and MacLeod M** (2001) A LIFE-ICZM demonstration project for Irish beaches and sand dunes. In *Coastal Dune Management*, University of Liverpool, , 24–33.

**Prie, AJ, Miller DL and Branch LC** (2008) Identification of structural and spatial features that influence storm-related dune erosion along a barrier-island ecosystem in the Gulf of Mexico. *Journal of Coastal Research*, **24**(4 SUPPL.), 168–175. doi:10.2112/06-0799.1.

**Reis CS, Freitas H and Antunes do Carmo JS** (2005) Leirosa sand dunes: a case study on coastal protection. In *Proceedings of the IMAM-Maritime Transportation and Exploitation of Ocean and Coastal Resources, Lisboa*, , 26–30.

**Seabloom EW, Ruggiero P, Hacke, SD, Mul, J and Zarnetske P** (2013) Invasive grasses, climate change, and exposure to storm-wave overtopping in coastal dune ecosystems. *Global Change Biology*, **19**(3), 824–832. doi:10.1111/gcb.12078.

**Silva R, Martínez ML, Odériz I, Mendoza E and Feagin RA** (2016) Response of vegetated dune-beach systems to storm conditions. *Coastal Engineering*, **109**, 53–62. doi:10.1016/j.coastaleng.2015.12.007.

**Splinter KD and Palmsten ML** (2012) Modeling dune response to an East Coast Low. *Marine Geology*, **329**–**331**, 46–57. doi:10.1016/j.margeo.2012.09.005.

**Unguendoli S, Biolchi LG, Aguzzi M, Pillai UPA, Alessandri J and Valentini A** (2023) A modeling application of integrated nature based solutions (NBS) for coastal erosion and flooding mitigation in the Emilia-Romagna coastline (Northeast Italy). *Science of The Total Environment*, **867**(161357), 1–21. doi:https://doi.org/10.1016/j.scitotenv.2022.161357.
