# Supplementary material for: The dilemma of coastal management: Exploitation or conservation?
Source: Camb Prism Coast Futur. 2024 Aug 14;2:e10. doi: 10.1017/cft.2024.10 (PMC12337577; doi:10.1017/cft.2024.10)
Supplement: Peer review history

# Editor report: Recommendation: The dilemma of coastal management: Exploitation or conservation? — R0/PR2

**DOI:** 10.1017/cft.2024.10.pr2

**Affiliations:** University College Cork, Ireland

Incorporate the references (possibly as a supplementary table) of the studies that were engaged with in both topics and presented in Figures 2 and 4.

If possible, broaden the appeal of the article a little by expounding on some other case studies or examples.

Give more consideration to solutions and potential ways forward - can this be attempted using, for example, lessons from the case studies / examples presented? Otherwise there is a risk the paper is a review of other work and not contributing to the literature.

# Editor report: Decision: The dilemma of coastal management: Exploitation or conservation? — R0/PR3

**DOI:** 10.1017/cft.2024.10.pr3

**Affiliations:** Geography, Cambridge University, United Kingdom of Great Britain and Northern Ireland

No accompanying comment.

# Editor report: Recommendation: The dilemma of coastal management: Exploitation or conservation? — R1/PR5

**DOI:** 10.1017/cft.2024.10.pr5

**Affiliations:** University College Cork, Ireland

No accompanying comment.

# Editor report: Decision: The dilemma of coastal management: Exploitation or conservation? — R1/PR6

**DOI:** 10.1017/cft.2024.10.pr6

**Affiliations:** Geography, Cambridge University, United Kingdom of Great Britain and Northern Ireland

No accompanying comment.

# Editor report: Recommendation: The dilemma of coastal management: Exploitation or conservation? — R2/PR8

**DOI:** 10.1017/cft.2024.10.pr8

**Affiliations:** University College Cork, Ireland

All comments have been addressed

# Editor report: Decision: The dilemma of coastal management: Exploitation or conservation? — R2/PR9

**DOI:** 10.1017/cft.2024.10.pr9

**Affiliations:** Geography, Cambridge University, United Kingdom of Great Britain and Northern Ireland

No accompanying comment.
